# Supplementary material for: Formation and Stability of Prebiotically Relevant Vesicular Systems in Terrestrial Geothermal Environments
Source: Life (Basel). 2017 Nov 30;7(4):51. doi: 10.3390/life7040051 (PMC5745564; doi:10.3390/life7040051)
Supplement: Supplementary file 1 [file life-07-00051-s001.pdf]

Table S1. Collection of water samples from hot springs at Ladakh.

Name of the hot spring	Latitude	Longitude	Elevation (in m, asl)	Temperature (in °C)	pH (at site of collection)	pH (in lab at 21°C)
Puga	33°23′12″	78°35′14″	4414	70.2	7.4	8.48
Chumathang	33°36′02″	78°32′42″	3944	74.1	8.72	8.64
Panamic	34°59′44″	77°41′31″	3206	73.5	7.58	8.37

Note: The difference in the pH measured at the site of collection and that in the laboratory is due to temperature at which it was measured.

Table S2. Fatty acids and their derivatives used in the experiment.

Fatty acid system	Combination of fatty acid and its derivative	Concentration of fatty acid	Concentration of derivative		Total concentration of the mixture	Ratio of fatty acid to its derivative
			Fatty alcohol	Fatty glyceride		
**Oleic acid system (C18:1)**	Only OA	6 mM	-	-	6 mM	-
	OA + OOH	4 mM	2 mM	-		2:1
	OA + GMO	4 mM	-	2 mM		2:1
	OA + OOH + GMO	4 mM	1 mM	1 mM		4:1:1
**10-Undecenoic acid system (C11:1)**	Only UDA	90 mM	-	-	90 mM	-
	UDA + UDOH	60 mM	30 mM	-		2:1
	UDA + UDG	60 mM	-	30 mM		2:1
	UDA + UDOH + UDG	60 mM	15 mM	15 mM		4:1:1
**Decanoic acid system (C10:0)**	Only DA	60 mM	-	-	60 mM	-
	DA + DOH	40 mM	20 mM	-		2:1
	DA + GMD	40 mM	-	20 mM		2:1
	DA + DOH + GMD	40 mM	10 mM	10 mM		4:1:1

Note: The total concentration of a particular fatty acid system was selected to be sufficiently above its CVC value.

Abbreviations of fatty acids and their derivatives with the respective chemical structures:

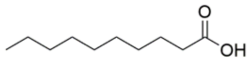

Decanoic acid (DA)

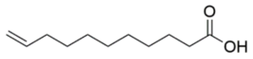

10-Undecenoic acid (UDA)

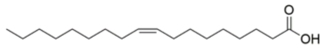

Oleic acid (OA)

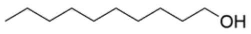

Decanol (DOH)

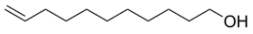

Undecenol alcohol (UDOH)

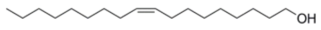

Oleyl alcohol (OOH)

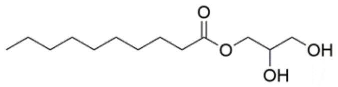

1-Decanoyl-rac-glycerol (GMD)

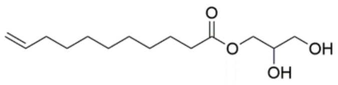

Monoundecenoin glyceride (UDG)

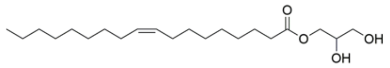

Monoolein (GMO)

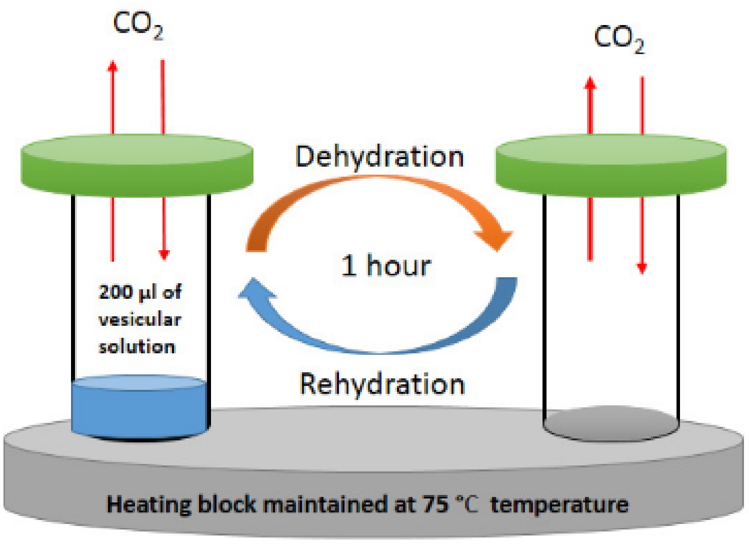

Figure S1. Schematic of DH-RH experiment. A vesicle solution is kept on the heating block with a constant CO_2 flow. During dehydration (DH), all water evaporates leaving behind a multilamellar sheet of amphiphiles, which again spontaneously forms vesicles upon rehydration (RH).

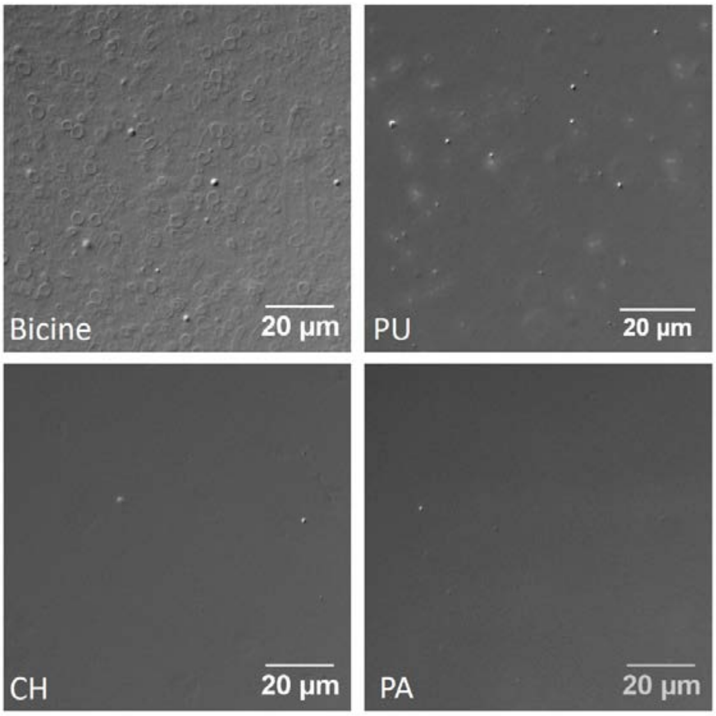

Figure S2. Formation of vesicles by OA. Vesicles were observed in 200 mM bicine buffer pH 8.5 (positive control), but not in Puga (PU), Chumathang (CH) or Panamic (PA).

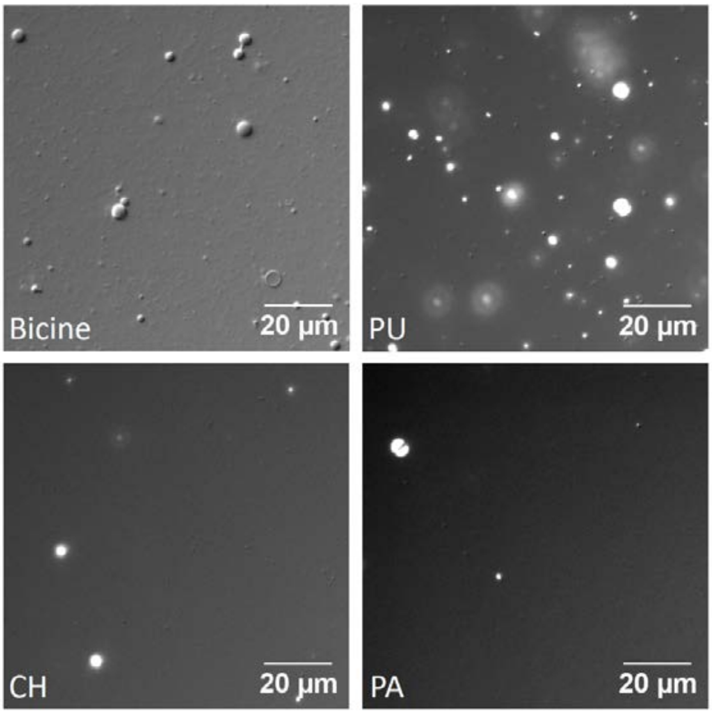

Figure S3. Formation of vesicles by OA and OOH binary system. Vesicles were present in 200 mM bicine buffer pH 8.5. However, only shiny oil droplets were observed in PU, CH and PA.

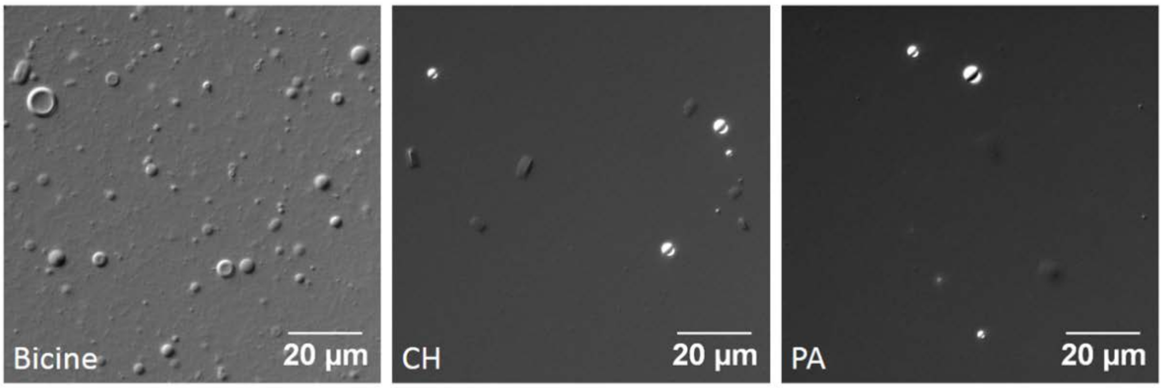

Figure S4. Formation of mixed fatty acid vesicles from the tertiary system comprising of OA, OOH and GMO. Vesicles were present in 200 mM bicine buffer pH 8.5 but not in CH and PA.

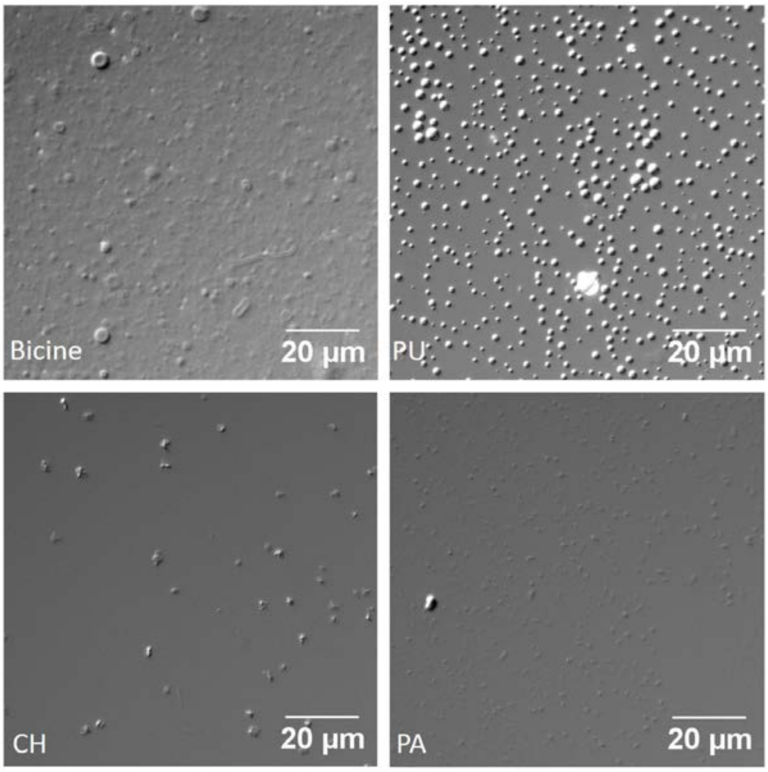

Figure S5. Formation of vesicles by UDA. Vesicles were present in 200 mM bicine buffer pH 8, but not in any of the hot spring water samples tested.

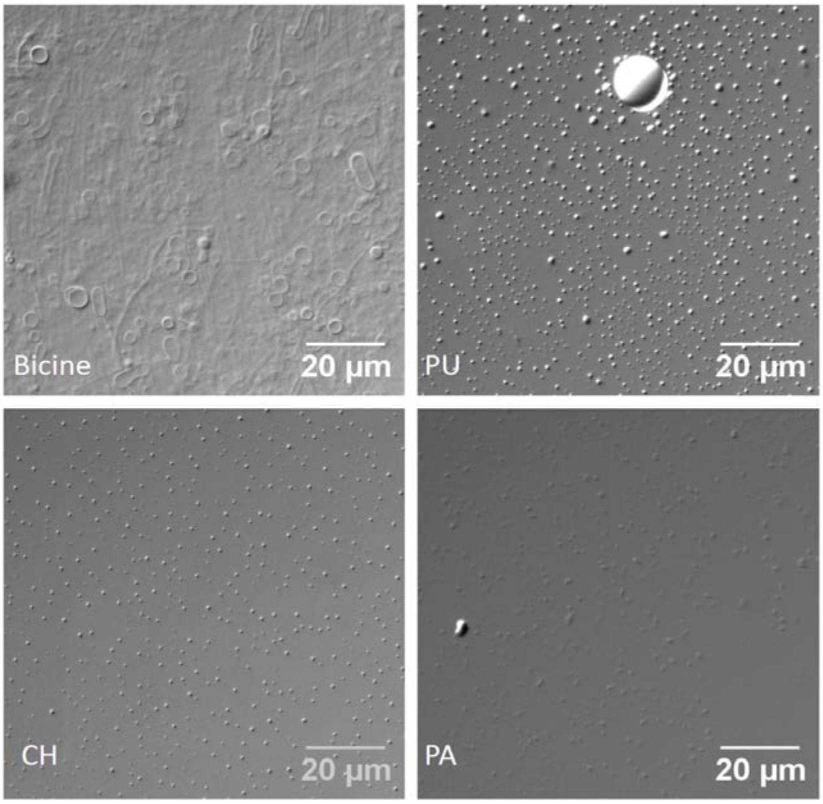

Figure S6. Formation of vesicles by UDA and UDOH. Vesicles were present in 200 mM bicine buffer pH 8, but not in any of the hot spring water samples that were tested.

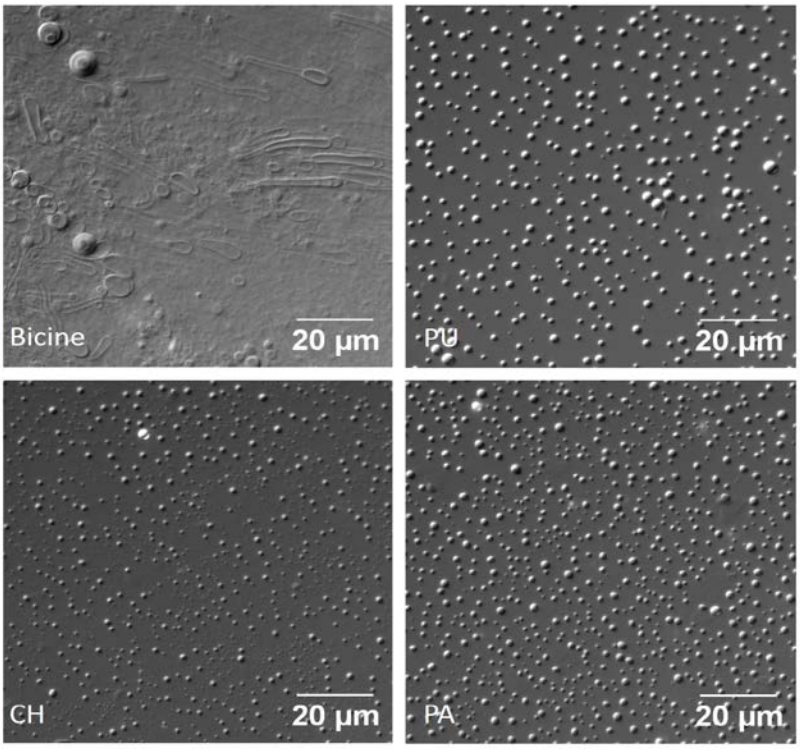

Figure S7. Testing the formation of mixed fatty acid vesicles by UDA, UDOH and UDG. Vesicles were present in 200 mM bicine buffer pH 8. Only droplets were observed in the hot spring samples tested.

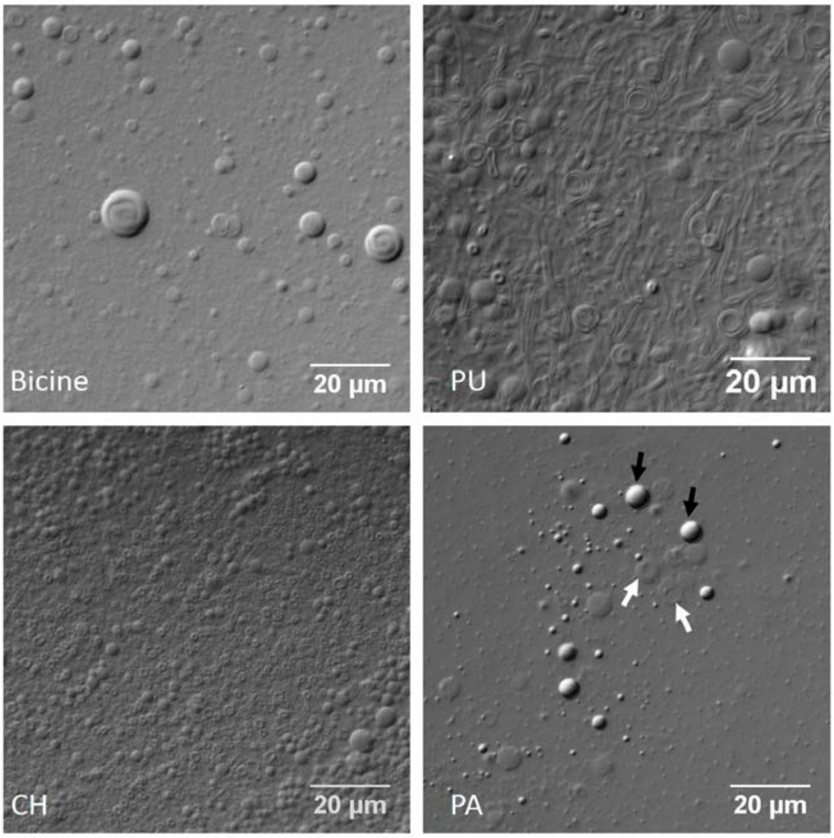

Figure S8. Formation of vesicles by UDA and UDG. Vesicles were present in 200 mM bicine buffer pH 8 and also in PU and CH. There were oil droplets in PA, but 3 hours of heating at 75^{0}C induced vesicle formation. The above image from PA shows a mixture of droplets (black arrows) and vesicles (white arrows) observed after heating.

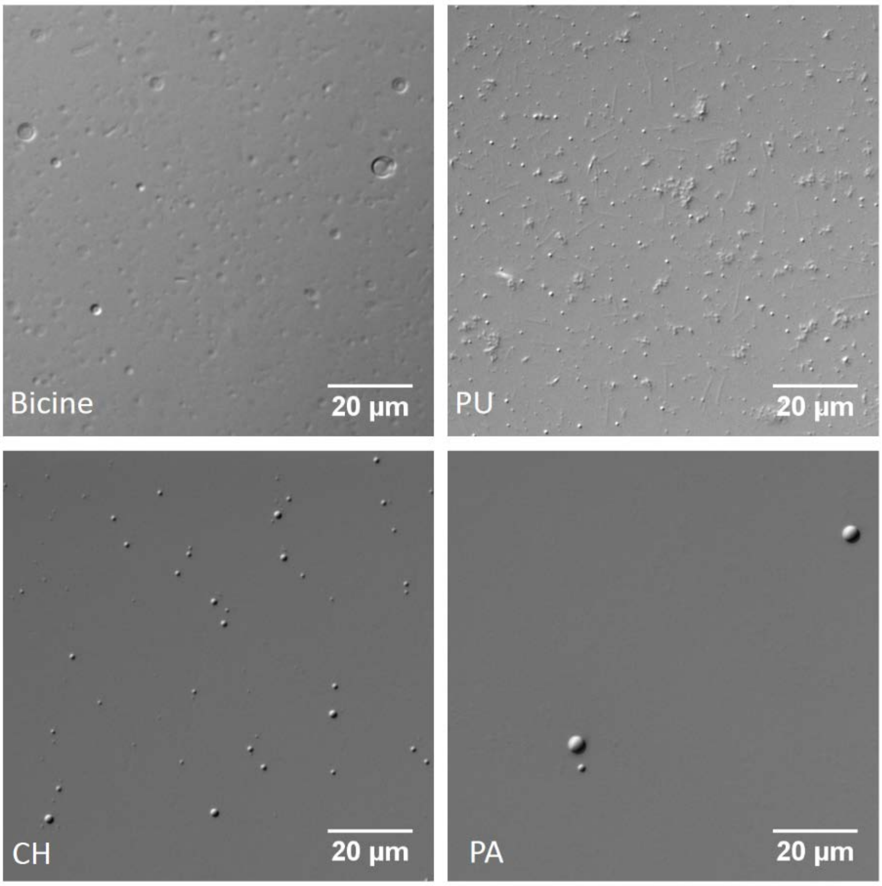

Figure S9. Formation of vesicles by DA. Vesicles were present in 200 mM bicine buffer pH 8, but not in any of the hot spring water samples that were tested.

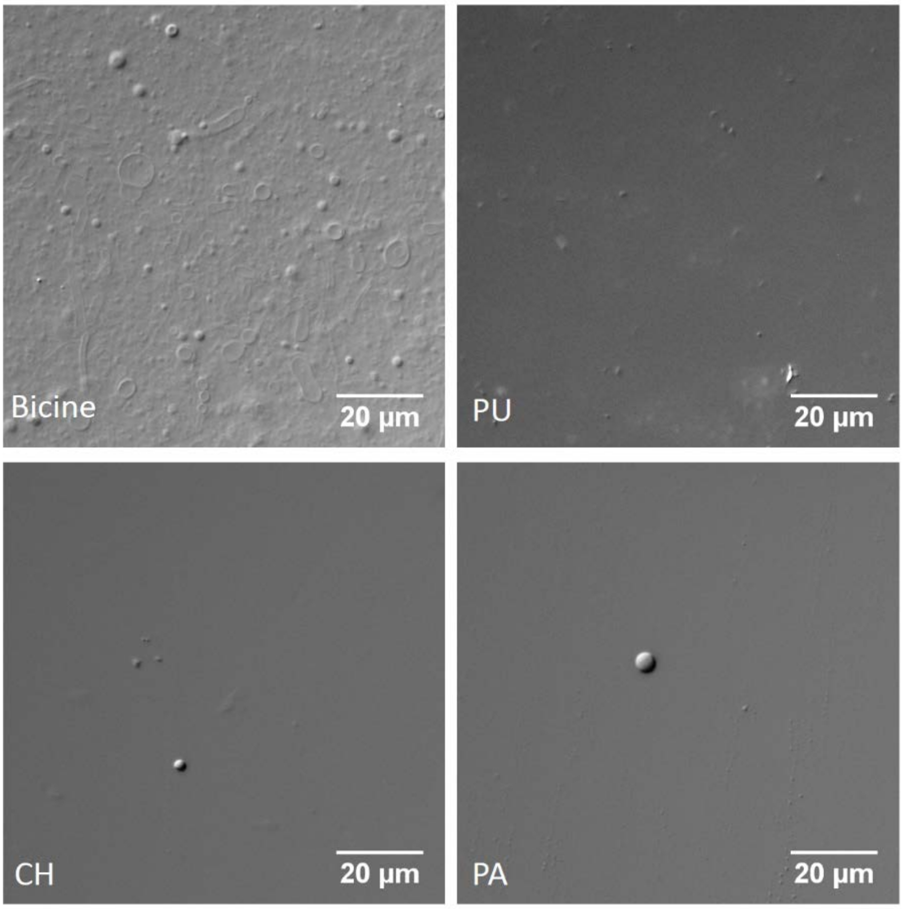

Figure S10. Formation of vesicles by DA and DOH. Vesicles were present in 200 mM bicine buffer pH 8 but not in any of the hot spring water samples that were tested.

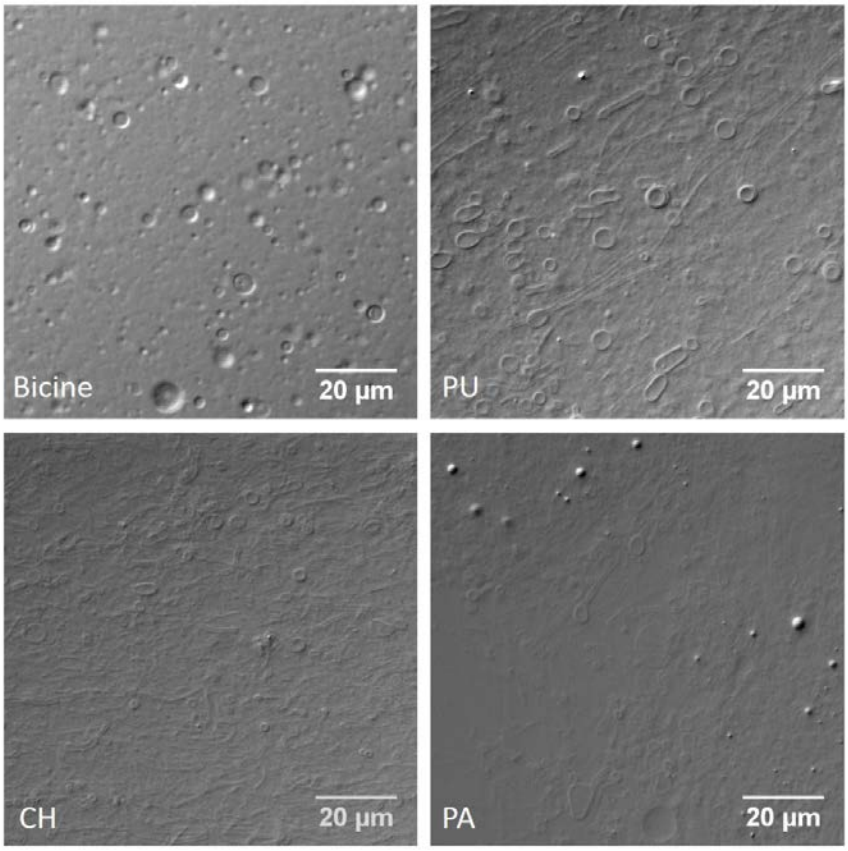

Figure S11. Formation of vesicles by DA and GMD. Vesicles were present in 200 mM bicine buffer pH 8 as well as in all the hot spring samples that were tested.

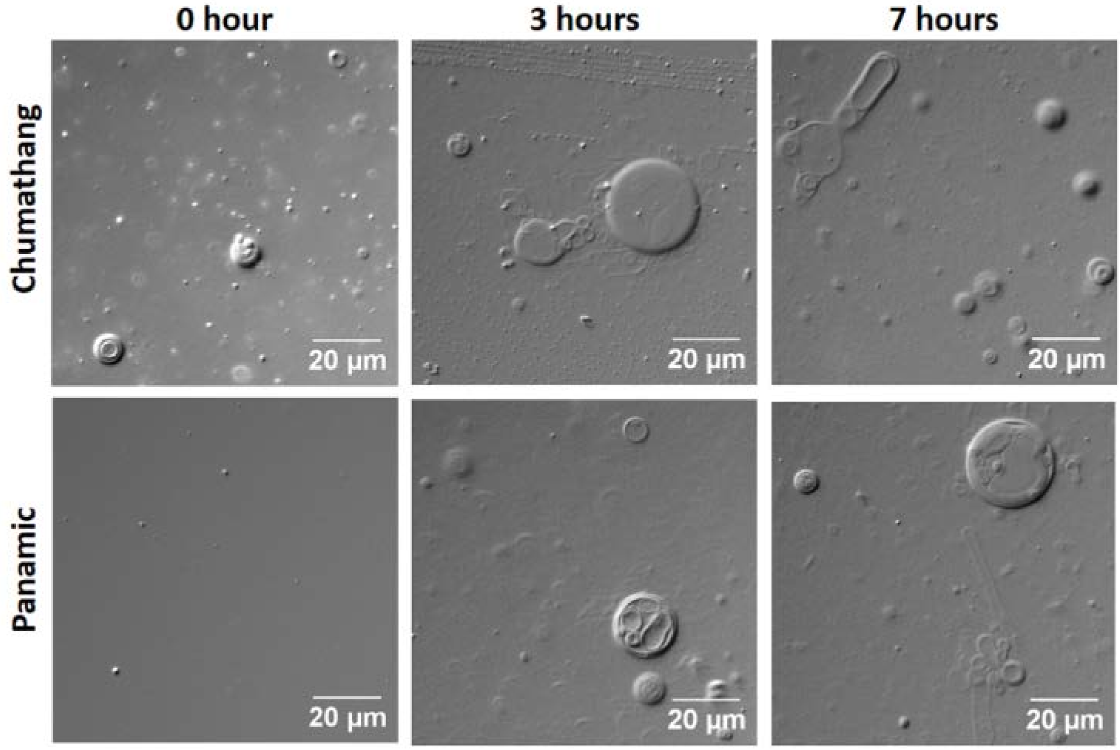

Figure S12. Temperature stability of OA and GMO mixed vesicles in CH and PA. Vesicles formed in the CH system and were stable up to 7 hours. However, in the PA system, vesicles formed only after 3 hours of heating and were stable up to 7 hours (lower panel images).

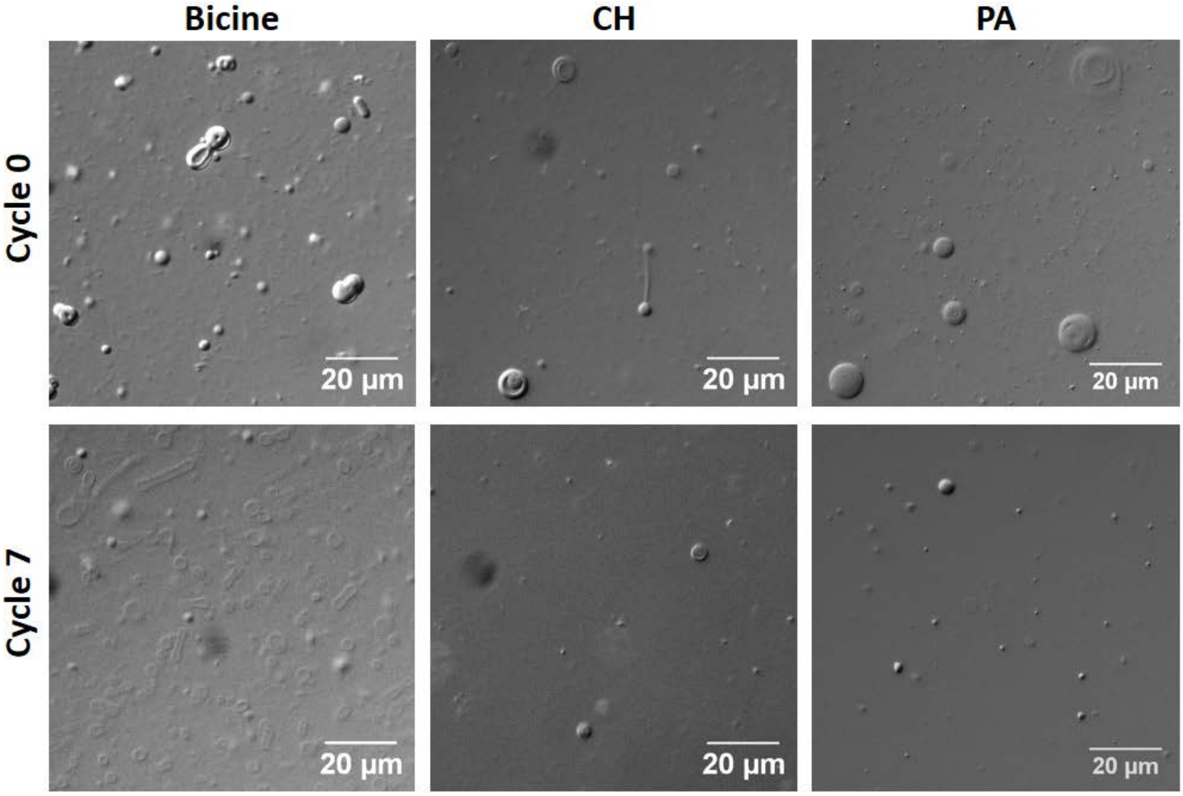

Figure S13. Stability of OA and GMO mixed vesicles in CH and PA under DH-RH conditions. Vesicles were observed in, both, the control sample and the hot spring water sample from CH even after 7 cycles of DH-RH. However, there was a visible reduction in the number and the size of the vesicles seen in CH. In case of PA water sample only droplets were mostly observed after 7 cycles of DH-RH.

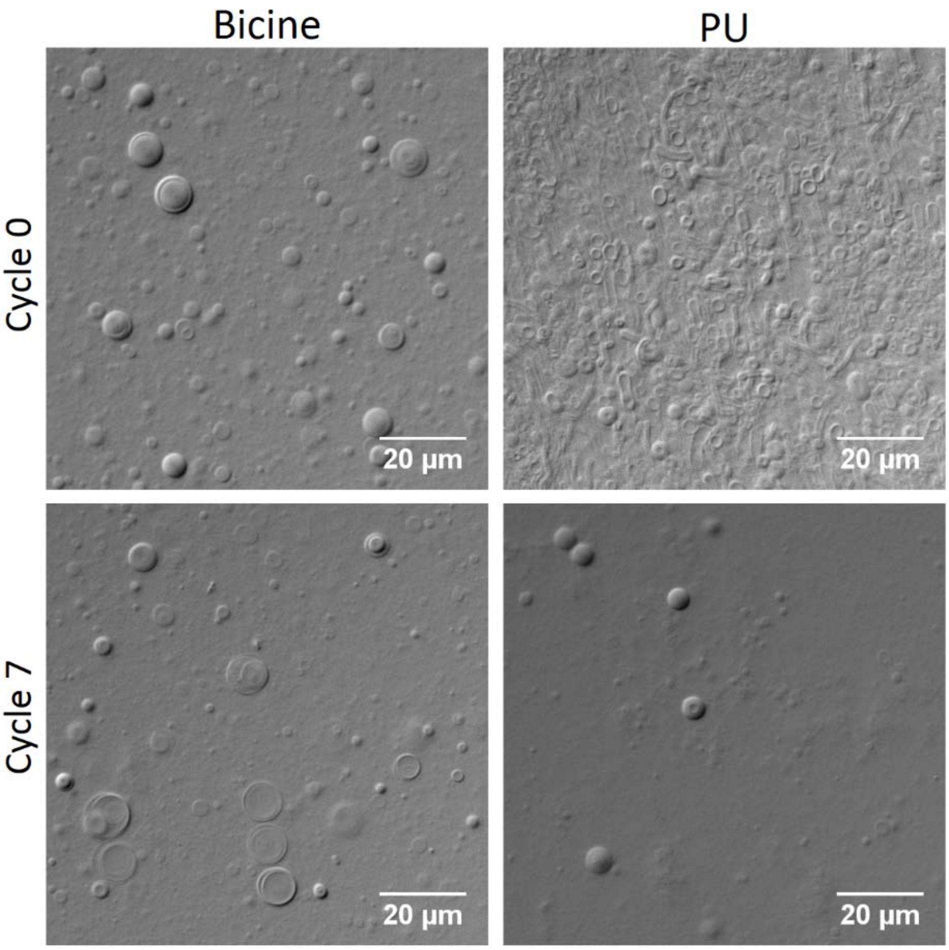

Figure S14. Stability of UDA and UDG mixed vesicles in PU under DH-RH conditions. Vesicles were observed even after 7 cycles of DH-RH.

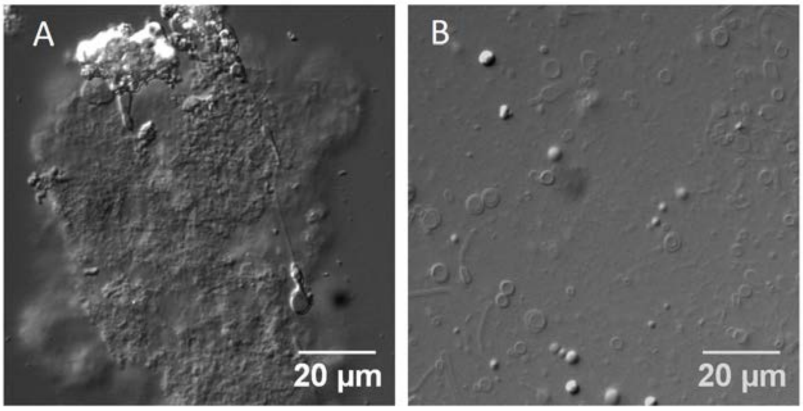

Figure S15. Effect of rehydration with bicine buffer on vesicle stability. Rehydration with bicine caused a two-fold increase of Na^+ concentration in the solution with every cycle of DH-RH, resulting in vesicle aggregation after cycle 5 (panel A). However, this effect was not observed when rehydration was performed with milli-Q water, and vesicles were quite stable after five DH-RH cycles (panel B).
